# Epigenetic Age Prediction Using N6‐Methyladenine in the Buff‐Tailed Bumblebee (*Bombus terrestris*)

**DOI:** 10.1111/acel.70312

**Published:** 2025-12-04

**Authors:** Thibaut Renard, Morgane Boseret, Serge Aron

**Affiliations:** ^1^ Evolutionary Biology and Ecology Université Libre de Bruxelles Brussels Belgium

**Keywords:** aging, DNA methylation, epigenetics, N6‐methyladenine

## Abstract

Epigenetic clocks are machine learning models that predict an organism's chronological age (the time elapsed since birth) or biological age (a proxy for physiological integrity) based on methylation levels from multiple genomic sites. To date, all epigenetic clocks rely exclusively on C5‐methylcytosine (5 mC), the predominant DNA methylation mark in vertebrates. However, not all species possess detectable 5 mC levels. Here, we used N6‐methyladenine (6 mA), a less‐characterized DNA modification type, to develop a series of epigenetic clocks in the buff‐tailed bumblebee ( *Bombus terrestris* ). Using long‐read Nanopore sequencing, we generated genome‐wide, base‐resolution profiles of 6 mA and 5 mC in males of different ages (*n* = 15), and developed multiple epigenetic clocks based on distinct features of the aging DNA methylome. All clocks showed strong correlations between predicted epigenetic and chronological age. Moreover, they also detected pharmacologically induced lifespan extension, reflected by a reduction in predicted epigenetic age relative to chronological age, indicating that these clocks capture biological aging. These findings demonstrate that 6 mA can be used to build accurate epigenetic clocks and establish 6 mA as a promising biomarker of aging in animals.

## Graphical abstract

In bumblebees, age‐related patterns of N6‐methyladenine can be used to build accurate epigenetic clocks that are responsive to lifespan‐extending interventions, providing the first proof of concept that this epigenetic mark can be used as a biomarker of aging.

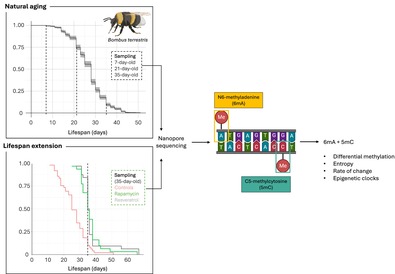

## 1 Introduction

Aging is a natural process characterized by a time‐dependent decline in biological integrity and functions, which increases the risk of intrinsic mortality and ultimately leads to death (Cohen 2018; López‐Otín et al. 2013). Epigenetic alterations are a hallmark of aging (López‐Otín et al. 2013). Age‐related epigenetic alterations can modify transcriptional patterns and thus disrupt cellular and physiological homeostasis, leading to biological aging (Lu, Tian, and Sinclair 2023). Thus, altering the epigenome can influence the aging process. For example, in mammals, restoring pristine epigenetic patterns can promote in vivo rejuvenation by “resetting” transcriptomic profiles and cellular functions (Chondronasiou et al. 2022; Lu et al. 2020; Yang et al. 2023).

DNA methylation (DNAm) has been consistently linked to the aging process (Horvath and Raj 2018; Seale et al. 2022). During aging, the DNA methylome experiences two contrasting types of alterations (stochastic vs. predictable) that contribute to age‐related phenotypes. Stochastic changes accumulate randomly across the genome over time, increasing epigenetic entropy and transcriptional noise, which can disrupt cellular function (Lu, Tian, and Sinclair 2023; Yang et al. 2023). In contrast, predictable changes occur consistently at specific genomic loci and can be leveraged to build epigenetic age prediction models (epigenetic clocks) which quantitatively predict chronological age (the actual time since birth) and/or biological age (an estimate of biological function) (Hannum et al. 2013; Horvath and Raj 2018). Epigenetic clocks have been primarily developed in mammals (Lu, Fei, et al. 2023) and, to a lesser extent, in amphibians (Zoller et al. 2024), fishes (Anastasiadi and Piferrer 2020), crustaceans (Hearn et al. 2021), and insects (Brink et al. 2024). Remarkably, it is possible to develop epigenetic clocks that use the same algorithm to predict epigenetic age in different species (Lu, Fei, et al. 2023), indicating that some features of epigenetic aging are evolutionarily conserved. To date, all existing epigenetic clocks developed rely exclusively on C5‐methylcytosine (5 mC), the predominant form of DNA methylation in eukaryotes (Zemach et al. 2010). However, in some taxa, 5 mC is extremely low (e.g., bees (Bewick et al. 2016) and fruit flies (Lyko et al. 2000)) or even undetectable (e.g., nematodes (Wenzel et al. 2011) and yeasts (Capuano et al. 2014)), raising the question of whether alternative epigenetic marks could serve as surrogates of aging biomarkers in these species.

N6‐methyladenine (6 mA) is a distinct type of DNAm, originally thought to be exclusive to prokaryotes where it is involved in cellular immunity (Varma et al. 2022). Recently, 6 mA has been identified in various eukaryotes, including *Chlamydomonas* (Fu et al. 2015), *C. elegans* (Greer et al. 2015), *D. melanogaster* (Zhang et al. 2015), *X. laevis* (Koziol et al. 2016), mice (Koziol et al. 2016), and humans (Koziol et al. 2016), where it is involved in gene expression regulation. Importantly, similarly to 5 mC, 6 mA is stably maintained and inherited in eukaryotes (Y. Wang et al. 2019, 2025), which is an essential prerequisite for its potential use as an epigenetic biomarker of aging. To date, the utility of 6 mA as an epigenetic biomarker of aging remains unexplored.

Here, we developed a series of epigenetic clocks based on 6 mA in the buff‐tailed bumblebee ( *Bombus terrestris* ). This species was used as a model system because its genome contains low levels of both 6 mA and 5 mC. We performed long‐read Oxford Nanopore Technologies sequencing on *B. terrestris* males of various ages (*n* = 15), covering their entire lifespan, to generate genome‐wide, base‐resolution 6 mA and 5 mC levels. First, we compared age‐associated patterns in 6 mA and 5 mC using differential methylation analyses, methylation entropy, and rate of change quantification. Second, we used age‐related patterns in 6 mA and 5 mC levels to generate highly accurate epigenetic clocks. We found that our 6 mA clocks can predict chronological age as accurately as 5 mC clocks. Third, we pharmacologically extended male lifespan and demonstrated that both 6 mA‐ and 5 mC‐based clocks yielded younger predictions of epigenetic age in treated males, indicating their ability to track biological aging. These findings provide the first proof of concept that 6 mA clocks can be as effective as 5 mC clocks in predicting both chronological and biological age in *B. terrestris* , paving the way toward their use among animals.

## 2 Results

### 2.1 Aging DNA Methylome

DNA methylation patterns undergo a collection of conserved age‐related alterations, including global hypomethylation, site‐specific hypermethylation, increased methylation variability (entropy), and linear shifts in methylation levels over time (Booth and Brunet 2016; Hannum et al. 2013; Horvath 2013). While these patterns have been extensively characterized for 5 mC, whether they also occur for 6 mA remains unknown. To test this, we sequenced the entire DNA methylome of *B. terrestris* males using long‐read Oxford Nanopore Technologies (ONT) sequencing to generate genome‐wide, base‐resolution levels of 6 mA and 5 mC in *B. terrestris* males. Three discrete age groups were selected (7, 21, or 35‐day‐old) to cover the entire lifespan of *B. terrestris* adult males (*n* = 5 per age group).

**FIGURE 1:**
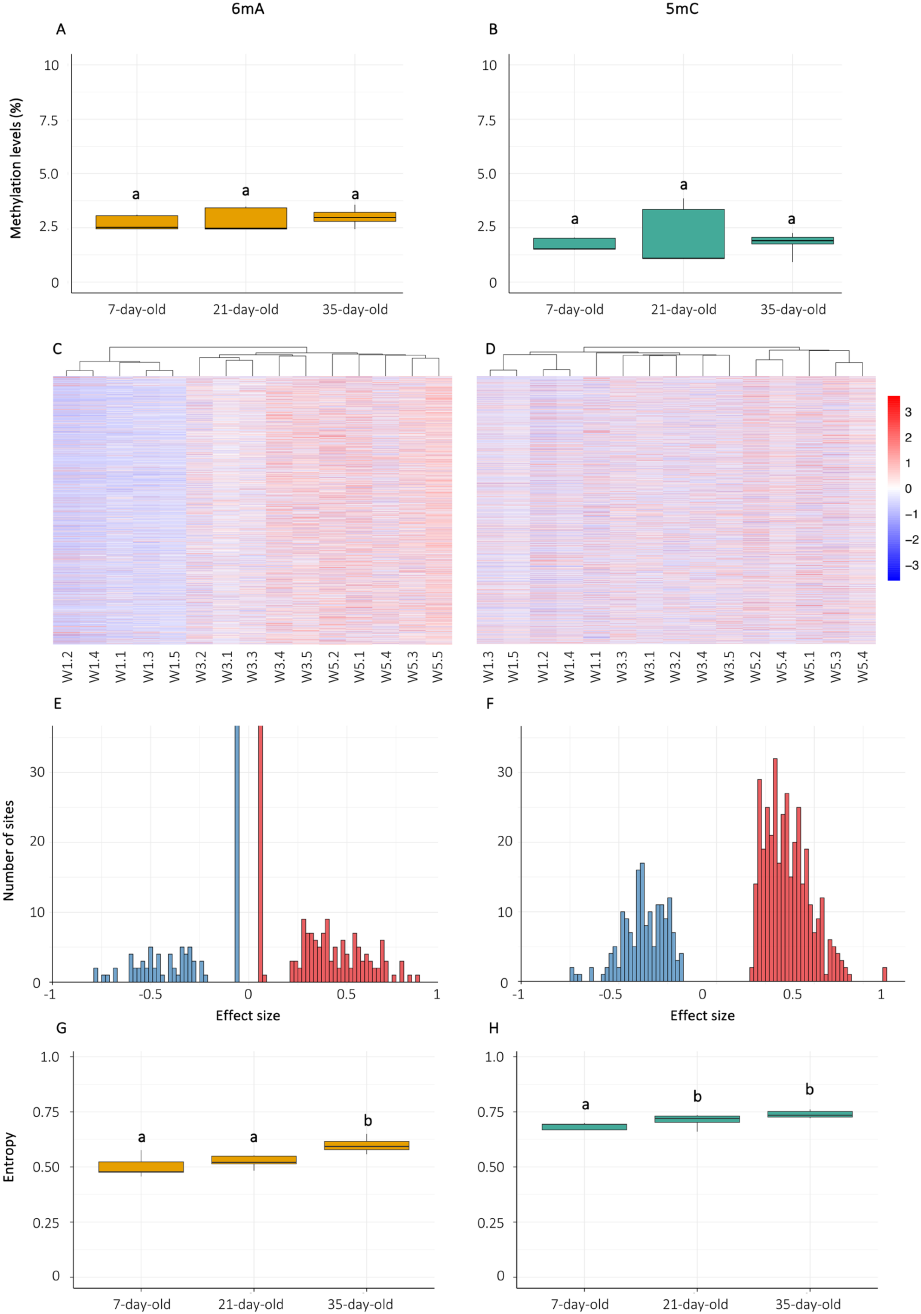
Age‐related changes in 6 mA and 5 mC in *Bombus terrestris* males. (A) Global levels of 6 mA and (B) 5 mC measured across the genome in 7‐day‐old, 21‐day‐old, and 35‐day‐old males. (C, D) Heatmaps with hierarchical clustering showing base‐resolution methylation levels of 6 mA or 5 mC at differentially methylated sites (DMS), respectively. Methylation levels were normalized per site using *Z*‐score transformation to allow relative comparison between age groups (W1 = 7‐day‐old, W3 = 21‐day‐old, W5 = 35‐day‐old). Blue indicates below‐average and red indicates above‐average methylation relative to the mean. (E, F) Distribution of effect sizes for 6 mA and 5 mC DMS between 35‐day‐old and 7‐day‐old (W1) males. Effect size is defined as the difference in mean methylation level between 35‐day‐old and 7‐day‐old individuals, such that positive values indicate hypermethylation and negative values indicate hypomethylation in older males. (G‐H) Average methylation entropy at 6 mA and 5 mC DMS across age groups. Statistical significance is denoted by different letters above the boxplots (*p* < 0.05). *n* = 5 per age group.

First, we investigated whether shifts in global methylation levels occur for 6 mA and/or 5 mC by quantifying global levels of each methylation type. Global levels of 6 mA were consistently higher than 5 mC across the three age groups (mean percentage of methylated sites ± SD: 6 mA = 2.85% ± 0.44% and 5 mC = 1.87% ± 0.83%, Wilcoxon Mann–Whitney *U* test: *U* = 199, *p* = 1.4e‐04). Neither methylation type experienced significant variation in global levels with age (mean global methylation levels ± SD: 6 mA: 7‐day‐old males = 2.71% ± 0.33%, 21‐day‐old males = 2.85% ± 0.55%, 35‐day‐old males = 2.99% ± 0.48%, Kruskal–Wallis test: chi‐squared = 0.06, df = 2, *p* = 0.97, Figure 1A; 5 mC: 7‐day‐old males = 1.72% ± 0.29%, 21‐day‐old males = 2.09% ± 1.39%, 35‐day‐old males = 1.79% ± 0.54%, Kruskal–Wallis test: chi‐squared = 0.14, df = 2, *p* = 0.93, Figure 1B), indicating that the DNA methylome does not undergo global hypomethylation in this species.

Second, we performed differential methylation analysis between 7‐day‐old and 35‐day‐old males to identify site‐specific alterations in the DNA methylome. Only extreme age groups were considered to capture the strongest age‐related changes. We identified 850 differentially methylated sites (DMS) for 6 mA and 530 DMS for 5 mC. Hierarchical clustering based on 6 mA or 5 mC DMS grouped individuals of the same age together (Figure 1C,D), indicating that they were more similar within each age group from a methylomic standpoint. The ratio of hypomethylated‐to‐hypermethylated sites differed between 6 mA and 5 mC: while most DMS for 6 mA showed increased methylation with age, the proportion of 5 mC that gained and lost methylation over time was more balanced (proportion of sites in 35‐day‐old vs. 7‐day‐old: 6 mA: 17.2% hypomethylated and 82.8% hypermethylated, Figure 1E, 5 mC: 30.9% hypomethylated and 69.1% hypermethylated, Figure 1F, chi‐squared test: chi‐squared = 34.736, df = 1, *p* = 3.775e‐09). Several DMS for 6 mA and 5 mC were found within noncoding RNAs (ncRNA). Specifically, 17.0% of 6 mA DMS were found in ncRNA and 82.0% in protein‐coding genes. In contrast, only 2.6% of 5 mC DMS occurred in ncRNAs, with the vast majority (97.4%) located in protein‐coding regions. This difference in distribution between 6 mA and 5 mC DMS was statistically significant (chi‐squared test: chi‐squared = 65.4, df = 1, *p* = 6.115e‐16). In addition, ~1% of 6 mA DMS were found in ribosomal DNA (rDNA), whereas no 5 mC DMS were detected in rDNA. The 6 mA DMS with the greatest methylation difference was located in the *TfIIFbeta* [*transcription factor TFIIFbeta*] gene, which encodes a subunit of the TFIIF transcription factor complex—an essential component of the RNA polymerase II preinitiation complex (Finkelstein et al. 1992). The cytosine displaying the largest methylation change was located in *RAPK2* (*rho‐associated protein kinase 2*), whose protein product is a serine–threonine kinase that regulates cell shape and movement by acting on the cytoskeleton (Amano et al. 2000). The complete list of 6 mA and 5 mC DMS can be found in ESM Tables S1 and S2. Functional enrichment analyses using gene ontology (GO) terms were performed to identify the biological processes (BP) associated with the aging DNA methylome of *B. terrestris* males. We identified 58 and 54 significantly enriched BP GO terms for 6 mA or 5 mC DMS, respectively. Enriched BP terms associated with 6 mA were involved in development, and epigenetic and epitranscriptomic regulation. Enriched 5 mC BP terms were mostly linked to proteostasis. The complete list of GO terms can be found in ESM Tables S3 and S4.

Third, we examined whether the *B. terrestris* DNA methylome undergoes stochastic age‐related changes in 6 mA and 5 mC. Shannon entropy was calculated for each 6 mA or 5 mC across the genome of each sample, then averaged across sites within the sample to derive an individual‐level measure of methylomic entropy (Hannum et al. 2013). Genome‐wide methylomic entropy did not undergo a significant increase with age for either methylation type (mean methylomic entropy ± SD: 6 mA: 7‐day‐old = 0.32 ± 0.02, 21‐day‐old = 0.34 ± 0.03, 35‐day‐old = 0.34 ± 0.03, one‐way ANOVA: *F* = 1.014, df = 2, *p* = 0.386, 5 mC: 7‐day‐old = 0.32 ± 0.02, 21‐day‐old = 0.34 ± 0.04, 35‐day‐old = 0.36 ± 0.04, one‐way ANOVA: *F* = 1.48, df = 2, *p* = 0.259). However, when considering DMS only, 6 mA and 5 mC methylomic entropy increased with age (6 mA: 7‐day‐old = 0.50 ± 0.05, 21‐day‐old = 0.52 ± 0.03, 35‐day‐old = 0.61 ± 0.03, one‐way ANOVA: *F* = 12.66, df = 2, *p* = 0.0006, post hoc Tukey HSD: 7‐day‐old vs. 35‐day‐old: *p* = 0.0009, 21‐day‐old vs. 35‐day‐old: *p* = 0.0072, Figure 1G, 5 mC: 7‐day‐old = 0.68 ± 0.02, 21‐day‐old = 0.71 ± 0.03, 35‐day‐old = 0.75 ± 0.01, one‐way ANOVA: *F* = 10.77, df = 2, *p* = 0.0013, post hoc Tukey HSD: 7‐day‐old vs. 35‐day‐old: *p* = 0.0010, Figure 1H).

### 2.2 Epigenetic Age Prediction

Next, we investigated whether linear changes in 6 mA and 5 mC occur within the aging DNA methylome of *B. terrestris* and, if so, whether they can be leveraged to build epigenetic age predictors (epigenetic clocks). To this end, we conducted a site‐specific rate of change (ROC) analysis to identify linear changes in 6 mA or 5 mC. For each DMS, we applied simple linear regression models to quantify the relationship between methylation levels and chronological age. Site‐specific ROC was measured by the model's slope, then averaged across significant models into mean ROC. Mean ROC was low for both methylation types. However, 6 mA ROC was significantly higher than 5 mC ROC (mean ROC per week ± SD [number of significant sites retained]: 6 mA = 1.32 ± 2.04% [*n* = 473], 5 mC = 0.61 ± 11.14% [*n* = 282], Wilcoxon rank‐sum test: *W* = 60.591, *p* = 0.035). In addition, the mean rate of change (ROC) in 5 mC methylation showed significantly greater variance compared to 6 mA (Levene's test for homogeneity of variance: *F* = 712.73, df = 1, *p* < 2.2e‐16). These results indicate that linear age‐related changes occur for both 6 mA and 5 mC, and that 5 mC DMS exhibit a broader range of methylation dynamics than 6 mA.

To explore whether linear changes in 6 mA or 5 mC levels can be leveraged to build epigenetic clocks, we applied penalized regression models to develop a series of clocks based on either 6 mA or 5 mC. We adopted a three‐step sequential approach to fully explore and exploit different features of the aging DNA methylome. Each model was trained using a leave‐one‐out cross‐validation (LOOCV) approach as an internal control, then tested on an independent (testing) dataset.

**FIGURE 2:**
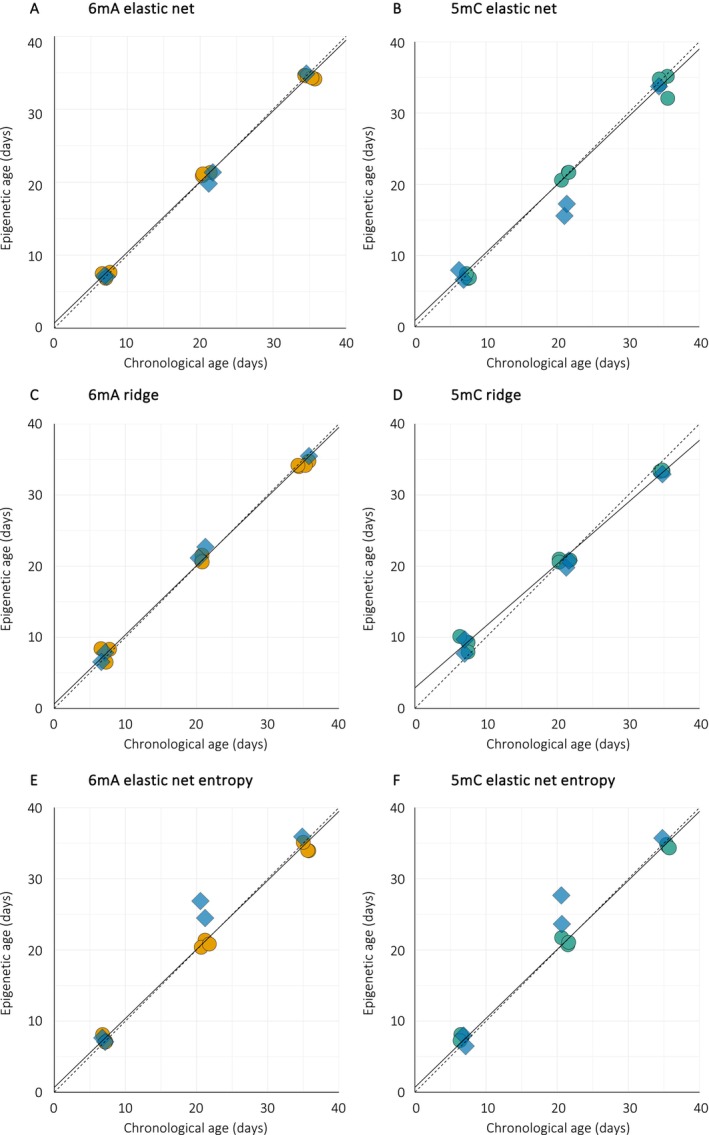
Epigenetic age prediction models in *Bombus terrestris* males. Epigenetic clocks were constructed using penalized regression models with leave‐one‐out cross‐validation (LOOCV), based on age‐associated patterns in 6 mA (A, C, E) or 5 mC (B, D, F). (A, B) Elastic net regression models were trained on genome‐wide methylation levels. (C, D) Ridge regression models were trained on methylation levels of differentially methylated sites (DMS). (E, F) Elastic net regression models were trained on entropy values computed at each DMS. Solid lines indicate the fitted epigenetic age prediction models. Dashed lines represent the identity line (y = x), showing the expected relationship under perfect age prediction. Data points represent individual samples: Orange (6 mA) and green (5 mC) circles correspond to training samples; blue diamonds represent test samples. *n* = 5 per age group.

The first type of epigenetic clocks was trained on methylation levels (6 mA or 5 mC). Elastic net regressions were used to identify sites that correlate with chronological age. During model training, elastic net regressions selected 48 adenine and 44 cytosine residues whose methylation levels strongly correlate with chronological age (training dataset: 6 mA clock: correlation = 0.999, mean relative error (MRE) = 2.6%, mean absolute error (MAE) = 0.3 days, *p* (linear model) < 2e‐16; 5 mC clock: correlation = 0.998, MRE = 3.1%, MAE = 0.5 days, *p* (linear model fitting) < 2e‐16). Among these, 9 adenine and 40 cytosine residues were located within coding regions. For the 6 mA clock, 3 sites were found in intergenic regions, with rDNA as the closest annotated sequence. Cytosines from the 5 mC clock were not significantly enriched in any biological process. When applied to the testing dataset, both 6 mA and 5 mC elastic net models accurately predicted chronological age (testing dataset: 6 mA clock: correlation = 0.987, mean relative error (MRE) = 7.3%, mean absolute error (MAE) = 1.8 days, Figure 2A; 5 mC clock: correlation = 0.978, MRE = 11.4%, MAE = 2.6 days, Figure 2B), despite slight underestimation of predicted age for 21‐day‐old males for the 5 mC clock.

The second type of epigenetic clocks was developed by using DMS only. Here, we used ridge regressions to build models using all 6 mA or 5 mC DMS as this type of penalized regression shrinks the coefficient of weakly predictive sites toward zero. As expected, model training reported very high age prediction accuracy (training dataset: 6 mA clock: correlation = 0.999, mean relative error (MRE) = 3.0%, mean absolute error (MAE) = 0.4 days, *p* (linear model) < 2e‐16; 5 mC clock: correlation = 0.9993, MRE = 10.6%, MAE = 1.3 days, *p* (linear model) = 6.9e‐13). Both 6 mA and 5 mC ridge models accurately predicted age in the naïve dataset (testing dataset: 6 mA clock: correlation = 0.985, mean relative error (MRE) = 5.4%, mean absolute error (MAE) = 1.6 days, Figure 2C; 5 mC clock: correlation = 0.9983, MRE = 10.7%, MAE = 1.3 days, Figure 2D).

Finally, for the third type of epigenetic clocks, we tested whether site‐specific methylation entropy could be used as a surrogate for methylation levels. While 5 mC entropy has recently been shown to correlate with chronological age (Chan et al. 2025), the potential use of 6 mA entropy remains unknown. Here, we used elastic net regression to select sites whose methylation entropy correlates with chronological age. Both entropy‐based clocks reported accurate performance metrics (training dataset: 6 mA clock: correlation = 0.9972, mean relative error (MRE) = 3.3%, mean absolute error (MAE) = 0.6 days, *p* (linear model) < 2e‐16; 5 mC clock: correlation = 0.9995, MRE = 3.1%, MAE = 0.5 days, *p* (linear model) < 2e‐16; testing dataset: 6 mA clock: correlation = 0.9528, mean relative error (MRE) = 11.4%, mean absolute error (MAE) = 2.6 days, Figure 2E; 5 mC clock: correlation = 0.9575, MRE = 10.4%, MAE = 2.3 days, Figure 2F). Elastic net regressions selected 37 adenines and 25 cytosines whose entropy methylation best predicted chronological age. Similarly to the 6 mA clock based on methylation levels, several adenine residues from the 6 mA entropy clock were located within or close to rDNA. Cytosines from the 5 mC entropy clock were found in genes involved in DNA replication, transcription and DNA damage repair.

### 2.3 Pharmacological Lifespan Extension and Biological Aging

Epigenetic clocks can be trained to predict either chronological or biological age. Our findings show that 6 mA and 5 mC correlate with chronological age in *B. terrestris* males. However, whether they are also associated with biological age remains to be tested. Since our experiments were conducted under standardized laboratory conditions, we did not expect significant interindividual variation in biological age between individuals. Therefore, we used pharmacological agents to increase individual lifespan, thus affecting biological age, and we tested whether the 6 mA‐ and 5 mC‐based clocks capture signals of biological aging.

**FIGURE 3:**
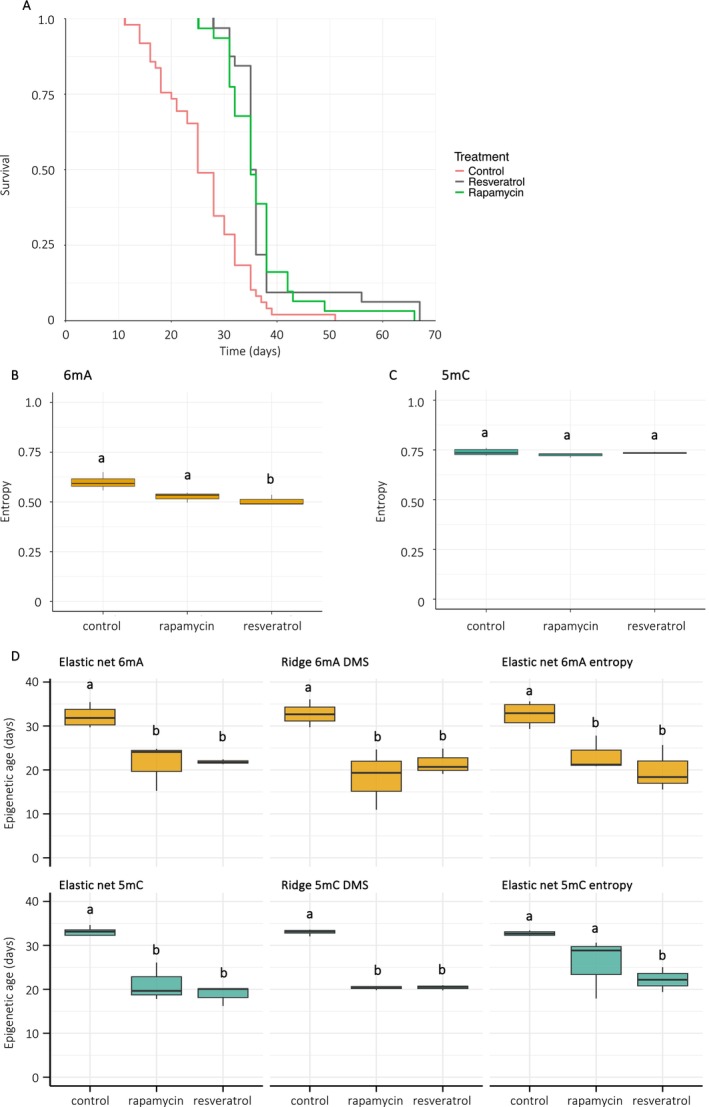
Pharmacological lifespan extension is associated with reduced epigenetic age in 35‐day‐old *B. terrestris* males. (A) Survival curves of males fed a control solution (light coral, *n* = 49), rapamycin (green, *n* = 32) or resveratrol (gray, *n* = 31) (Cox proportional hazards regression model: Rapamycin‐fed males: *p* = 6.61e‐07, resveratrol‐fed males: *p* = 8.98e‐07). (B, C) Entropy levels of 6 mA and 5 mC DMS, respectively (*n* = 3 per treatment group). (D) Predicted epigenetic ages of 35‐day‐old males fed rapamycin or resveratrol (*n* = 3 per treatment group). Different letters above the box represent significant statistical differences (*p* < 0.05) using either one‐way ANOVA and post hoc Tukey HSD test or Kruskal‐Wallis and post hoc Wilcoxon‐Mann–Whitney pairwise test.

First, we tested if bumblebee males' lifespan can be pharmacologically extended by chronically feeding them with rapamycin and resveratrol, two well‐researched agents which act upon the lifespan‐regulation mTOR (mechanistic target of rapamycin) and sirtuins pathways, respectively. The mean lifespans of males fed rapamycin and resveratrol were increased by 37% and 34%, respectively, compared to males fed a control solution containing sugar syrup and DMSO (mean lifespan ± SD: control = 26.9 ± 7.4 days, rapamycin = 37.6 ± 8.8 days, resveratrol = 36.5 ± 7.4 days, Kruskal‐Wallis: chi‐squared = 42.388, df = 2, *p* = 6.2e‐10, Figure 3A). Both treatments caused a 31% increase in maximum lifespan (control = 51 days, rapamycin = 67 days, resveratrol = 66 days) (Figure 3A).

Second, we investigated whether pharmacological lifespan extension is associated with methylomic changes. We sequenced the DNA methylome of 35‐day‐old rapamycin‐ or resveratrol‐fed males and compared it to 35‐day‐old control males. Global DNAm levels did not vary significantly between control males, rapamycin‐ and resveratrol‐fed males (6 mA: control = 3.37% ± 0.21%, rapamycin: 2.95% ± 0.42%, resveratrol: 2.88% ± 0.31%, one‐way ANOVA: *F* = 1.972, df = 2, *p* = 0.22, 5 mC: control = 1.84% ± 0.42%, rapamycin = 2.08% ± 0.26%, resveratrol = 2.07% ± 0.13%, one‐way ANOVA: *F* = 3.028, df = 2, *p* = 0.11). Differential methylation analyses revealed that both rapamycin and resveratrol had much stronger effects on 5 mC than 6 mA (6 mA DMS: rapamycin = 10, resveratrol = 4; 5 mC DMS: rapamycin = 14,745, resveratrol = 8081). Both treatments induced 5 mC DMS, but not 6 mA DMS, in genes related to their target protein (rapamycin: *raptor* [*regulatory associated protein of MTOR complex 1*], *lamtor* [*late endosomal/lysosomal adaptor, MAPK and MTOR activator*]; resveratrol: *sirt1* [*NAD + ‐dependent protein deacetylase sirtuin‐1*]) (ESM Tables S5 and S6). Functional enrichment analyses for 6 mA identified 37 and 6 enriched BP terms between rapamycin‐ or resveratrol‐fed versus control males. For 5 mC DMS, 277 and 283 BP terms were enriched between rapamycin or resveratrol males versus the controls, respectively. These included functions related to aging hallmarks, such as proteostasis, genomic maintenance, epigenetic regulation and autophagy (ESM Tables S7 and S8). Surprisingly, DMS‐specific 6 mA and 5 mC entropy remained stable across controls and treatments, except for 6 mA levels which were reduced in resveratrol‐fed males (mean methylomic entropy ± SD: 6 mA: control = 0.60 ± 0.03, rapamycin = 0.53 ± 0.03, resveratrol = 0.51 ± 0.03, Kruskal–Wallis: chi‐squared = 9.84, df = 2, *p* = 0.007, Wilcoxon–Mann–Whitney pairwise post hoc test: *p* = 0.02 between control vs. resveratrol; *p* > 0.05 for other comparisons; 5 mC: control = 0.74 ± 0.02, rapamycin = 0.72 ± 0.01, resveratrol = 0.73 ± 0.01, Kruskal–Wallis: chi‐squared = 1.42, df = 2, *p* = 0.49, Figure 3B,C).

Third, we investigated whether pharmacological lifespan extension is linked to reduced methylomic entropy. We assessed methylomic entropy in 35‐day‐old males treated with rapamycin or resveratrol and compared it to controls of the same age group. At the genome‐wide level, neither treatment significantly altered methylomic entropy (mean methylomic entropy ± SD: 6 mA: rapamycin = 0.32 ± 0.01, resveratrol = 0.35 ± 0.01, control = 0.34 ± 0.03, Kruskal‐Wallis: chi‐squared = 5.956, df = 2, *p* = 0.51; 5 mC: rapamycin = 0.36 ± 0.02, resveratrol = 0.38 ± 0.01, control = 0.33 ± 0.01, one‐way ANOVA: *F* = 1.06, df = 2, *p* = 0.54). However, when restricting the analysis to DMS only, we found a significant decrease in 6 mA entropy, but not 5 mC entropy (6 mA: rapamycin = 0.52 ± 0.03, resveratrol = 0.51 ± 0.03, control = 0.59 ± 0.04, Kruskal–Wallis: chi‐squared = 4.62, df = 2, *p* = 0.05; 5 mC: rapamycin = 0.72 ± 0.01, resveratrol = 0.73 ± 0.01, control = 0.72 ± 0.01, one‐way ANOVA: *F* = 1.861, df = 2, *p* = 0.25).

Finally, we evaluated whether the different epigenetic clocks capture signals of biological aging by comparing the predicted epigenetic age of treated versus control males of the same chronological age (35‐day‐old). Each clock predicted that the epigenetic age of at least one treatment group (rapamycin or resveratrol) was significantly lower than that of control males (Figure 3D). These results indicate that both treatments effectively decelerate epigenetic aging, and suggest that our epigenetic clocks track biological aging.

## 3 Discussion

The epigenetic clock theory of aging proposes that epigenetic age, as measured by epigenetic clocks, stems from unintended consequences of normal developmental and maintenance programs, thereby capturing intrinsic aging processes that are evolutionarily conserved (Horvath and Raj 2018). However, while all organisms undergo development to reach maturity, not all species possess detectable levels of 5 mC. This raises the possibility that other epigenetic marks may also contribute to aging regulation and could serve as alternative biomarkers of aging. To our knowledge, this study is the first to develop 6 mA epigenetic clocks that track both chronological and biological age in an animal, paving the way toward the use of this epigenetic mark as an aging biomarker in species with low or undetectable levels of 5 mC.

We identified significant changes in 6 mA and 5 mC in the DNA methylomes of *B. terrestris* males with age. Entropy and ROC analyses reveal that both stochastic and consistent linear alterations occur in the *B. terrestris* DNA methylome during aging, albeit at relatively low magnitudes. Interestingly, the average ROC for both 6 mA and 5 mC is comparable to the mean per‐site methylation change observed among the 353 CpG sites of the Horvath epigenetic clock (Horvath 2013), further supporting the idea that the pace of epigenetic aging may be evolutionarily conserved across animal taxa (Lu, Fei, et al. 2023).

We found that age‐related changes in 6 mA and 5 mC patterns are linked to processes associated with the hallmarks of aging, yet each mark is enriched in distinct biological pathways. For example, 6 mA marks are predominantly associated with biological processes related to development, suggesting they potentially play a role in regulating developmental processes. This finding aligns with previous results from *D. melanogaster* , where 6 mA is involved in tissue‐specific expression of developmental and regulatory genes (Shah et al. 2019). In contrast, 5 mC DMS are enriched in several pathways linked to proteostasis, whose age‐related alterations are a primary hallmark of aging (López‐Otín et al. 2013). The enrichment of 6 mA and 5 mC in distinct biological functions suggests that each methylation type captures different, complementary features of aging.

We show that 6 mA patterns correlate with chronological age by training epigenetic clocks on different features of the aging DNA methylome (methylation levels vs. entropy). Our analyses reveal that both 6 mA and 5 mC accurately correlate with chronological age in *B. terrestris* males. Sites used by the 6 mA‐ or 5 mC‐based clocks are located in genes involved in different biological processes. Notably, previous work has shown that 5 mC clocks track some hallmarks of aging, but not all (Kabacik et al. 2022). Thus, integrating 6 mA and 5 mC data may provide a more comprehensive and multidimensional view of aging, enhancing the biological relevance and interpretability of epigenetic clocks. In addition, some of the sites from our 6 mA clocks are found in or near rDNA. Previous work has reported that 5 mC epigenetic clocks based solely on rDNA can achieve comparable age prediction accuracy to clocks built from genome‐wide data (M. Wang and Lemos 2019). Therefore, 6 mA in rDNA represents a promising target for developing cost‐effective epigenetic clocks using targeted sequencing approaches.

Using pharmacological agents to modulate individual lifespan, we found that 6 mA‐based epigenetic clocks not only track chronological age but also capture signals of biological aging. We used rapamycin and resveratrol to significantly extend *B. terrestris* males' lifespan, further expanding the range of species whose longevity is positively influenced by these agents (Bonkowski and Sinclair 2016; Harrison et al. 2009; Rallis et al. 2013). At the DNA methylome level, both agents altered the methylation of a restricted number of adenines and a large number of cytosines, including some occurring within genes involved in key lifespan‐regulating pathways. These genes included those encoding the agents' target proteins (target of rapamycin (TOR) for rapamycin and NAD + ‐dependent protein deacetylase sirtuin‐1 (SIRT1) for resveratrol) which are highly conserved regulators of lifespan across numerous animal species (Blagosklonny 2013; Michan and Sinclair 2007; Wood et al. 2004). In addition, almost half of the enriched BP terms were common to rapamycin‐ and resveratrol‐fed males, indicating that these agents act upon similar biological pathways. Furthermore, several biological processes were enriched during natural aging (comparing 7‐day‐old vs. 35‐day‐old control males) and pharmacologically induced lifespan extension (comparing control 35‐day‐old males vs. 35‐day‐old males treated with rapamycin or resveratrol), indicating that these agents may extend lifespan by modulating pathways that are altered during normal aging. These findings reinforce the growing pool of evidence that aging may result in part from the persistence of molecular, cellular, and physiological processes that are advantageous early in life but become deleterious over time (Blagosklonny 2013). Therefore, it seems likely that lifespan‐extending interventions enhance longevity by acting upon the biological pathways that underlie the natural aging process. In line with this, based on the predictions of the 6 mA and 5 mC clocks, the methylomes of the 35‐day‐old rapamycin‐ or resveratrol‐fed males reflected a younger epigenetic age, a methylation state like that of chronologically younger individuals. This demonstrates that epigenetic clocks based on 6 mA and 5 mC similarly capture signals of biological aging and can detect interventions that slow the aging process.

In short, our study exploits 6 mA to construct multiple epigenetic clocks that accurately predict both chronological and biological age. This study has some limitations. First, the sample size was modest. However, unlike bisulfite sequencing which detects only 5 mC (mainly in CpG contexts), ONT sequencing enables comprehensive methylation profiling across genomic contexts and methylation types. This substantially increases the number of potentially informative sites for age prediction. Second, the large number of sites raises the possibility of overfitting age to false positives within the training dataset. To limit this risk, we performed two independent external tests. First, we split the initial dataset into training and testing subsets to evaluate age predictions on samples not used for model training. Second, in the pharmacological experiment, we confirmed clock performance using untreated control samples. In both cases, predicted epigenetic ages correlated strongly with chronological age, indicating that the clocks rely on methylation patterns that generalize across independent datasets rather than on random associations arising from the high dimensionality of the data. Third, survivor bias (i.e., considering only individuals that have survived to a given timepoint) could lead to erroneously interpreting health‐associated sites as age‐associated sites in older (35‐day‐old) individuals. More finely resolved, time‐point‐specific sampling would help clarify and potentially resolve this issue. Finally, because we used commercially reared individuals, the generalizability of our findings should be further evaluated across other genetic backgrounds and environmental exposures. Despite these limitations, our study provides proof of concept that 6 mA can serve as a reliable biomarker of aging, paving the way for its use in species where 5 mC is present at low or undetectable levels.

## 4 Materials and Methods

### 4.1 Model Organism

The buff‐tailed bumblebee ( *Bombus terrestris* ) was used as a model system because (i) both 6 mA and 5 mC occur at very low levels in insect genomes (Bewick et al. 2016; Boulet et al. 2023), including that of *B. terrestris* (Renard et al. 2023), making this species ideal for studying both DNAm types simultaneously, (ii) its relatively small genome size (~393 Mb) (Crowley et al. 2023) makes this species suitable for cost‐effective Oxford Nanopore Technologies (ONT) sequencing, and (iii) we previously showed that the mean and maximum lifespan of *B. terrestris* workers can be experimentally extended using a single topical application of the pharmacological hypomethylating agent RG108, indicating that DNAm (5 mC) plays a role in lifespan regulation in this species (Renard et al. 2023).

*B. terrestris* males were obtained from Biobest (Westerlo, Belgium). A total of 150 males from 10 different colonies of origin were randomly assigned to 15 experimental microcolonies so that each contained 10 males. Microcolonies were maintained under standard laboratory conditions (red light, temperature = 27 ± 1°C, relative humidity = 50%–60%) and were provided with *ad libitum* access to sugar syrup (Biogluc, Biobest, Westerlo, Belgium). Preliminary survival experiments revealed that mean male lifespan in the lab is 27.4 ± 7.1 days (maximum = 51 days, *n* = 791). Therefore, we used males that were 7, 21, and 35 days old in our subsequent molecular analyses to represent the entire lifespan of bumblebee adult males. Since epigenetic age has been shown to oscillate during the day (Koncevičius et al. 2024), individuals were sampled at the same time of the day (10–11 AM) to prevent circadian‐related bias. Individuals were randomly sampled across microcolonies, flash frozen in liquid nitrogen, and stored at −80°C until further processing. In total, 15 bumblebee males were sequenced across the three age groups (*n* = 5 per age group).

### 4.2 Long‐Read Oxford Nanopore Technology Sequencing

Genome wide, base‐resolution DNA methylation levels were generated with long‐read ONT sequencing. High molecular weight genomic DNA (gDNA) was extracted from the thorax and legs of males 7, 21, or 35‐day‐old (*n* = 15) using an in‐house SDS/proteinase K protocol. The head and abdomen were discarded to exclude pheromonal head glands and the content of the digestive tract, respectively. Briefly, frozen tissues were individually dry ground, suspended in an SDS/proteinase K solution, and left in suspension overnight. Next, we performed phenol–chloroform/chloroform purification followed by gDNA precipitation using ethanol and sodium acetate. gDNA integrity was assessed using agarose gel electrophoresis. qDNA quantity and absorbance ratios were measured using a Qubit 3.0 fluorometer (Thermo Fisher Scientific) and a NanoDrop ONE spectrophotometer (Thermo Fisher Scientific), respectively. gDNA libraries were prepared with the Ligation Sequencing gDNA—Native Barcoding Kit 96 V14 (ONT, SQK‐NBD114.96) and sequenced with a PromethION platform (ONT, PRO‐SEQ002).

### 4.3 DNA Methylation Analyses

Raw reads were basecalled using Dorado (ONT, v. 0.7.2) with the super high accuracy model (“sup” command) to capture signals of modified bases (6 mA and 5 mC) in any genomic context. Basecalled reads aligned to the *B. terrestris* GCF_910591885.1 v. 1.2 reference genome (https://www.ncbi.nlm.nih.gov/datasets/genome/GCF_910591885.1/) using Dorado (“aligner” function). SAMtools was used for indexing, sorting and coverage quantification (mean coverage per sample ± SD = 32.6 ± 10.7X). Genome‐wide global methylation levels were generated based on aligned BAM files using Modkit (ONT, v. 0.3.2) “summary” command. Genome‐wide, base‐resolution methylation levels for 6 mA and 5 mC were generated using Modkit (ONT, v. 0.3.2) “pileup” command. Only sites with a minimum coverage of 10X were kept in the subsequent analyses. After coverage filtering, 85,228,908 ± 19,234,608 adenines and 146,060,972 ± 32,906,853 cytosines were conserved. All subsequent analyses were conducted separately for 6 mA and 5 mC. Differential methylation analysis was performed between 7‐day‐old and 35‐day‐old males using Modkit (ONT, v0.3.2) “dmr pair” function to identify significant age‐related differentially methylated sites (DMS) for 6 mA or 5 mC. Functional enrichment analyses using gene ontology (GO) terms were conducted by linking DMS with biological processes (BP) GO terms from Hymenoptera Genome Database (Walsh et al. 2022). Gene IDs were found at: www.ncbi.nlm.nih.gov/data‐hub/gene/taxon/30195/.

Methylation entropy and ROC were calculated as described in (Hannum et al. 2013). Briefly, site‐level methylation entropy was quantified and then averaged to sample‐level entropy. ROC analysis was performed by fitting simple linear models to age‐related changes in 6 mA or 5 mC levels. Significant models (*p* < 0.05 and *R* ^2^ > 0.5) were averaged to sample‐level ROC.

### 4.4 Epigenetic Clocks

Epigenetic age prediction models (epigenetic clocks) were developed using penalized regression models to identify sites whose methylation tracks chronological age. To avoid model overfitting and ensure generalizability, we randomly split our initial dataset (*n* = 15) into training (*n* = 10) and testing (*n* = 5) datasets. Sample splitting between training and testing datasets was consistent between each model to ensure comparability. Three types of epigenetic clocks were developed for each DNAm type (6 mA or 5 mC): elastic net regressions based on 6 mA or 5 mC levels, ridge regression based on 6 mA or 5 mC levels of significantly DMS, and elastic net regressions based on 6 mA or 5 mC entropy levels. While elastic net regressions (alpha = 0.5) perform feature selection to only retain sites that correlate highly with chronological age, ridge regressions (alpha = 0) retain all input sites but shrink the model coefficient of weakly predictive sites toward zero without driving them entirely to zero. For each model, a leave‐one‐out cross‐validation (LOOCV) approach was implemented to perform model training independently within *n* folds (i.e., 15 folds), which reduces the risk of overfitting. The LOOCV was nested into an outer loop for performance estimation and an inner loop for lambda hyper‐parameter fine‐tuning. Feature selection stability was assessed across LOOCV folds for each elastic net model (ridge models do not perform feature selection). The coefficient of variation for feature selection ranged between 6.9% and 15.5% (mean number of selected features during LOOCV: elastic net 6 mA = 49.3% ± 6.9%, elastic net 5 mC = 38.2% ± 7.9%, elastic net entropy 6 mA = 72.2% ± 9.6%, elastic net entropy 5 mC = 77.5% ± 15.5%), indicating stable feature selection in elastic net models. Permutation tests (*n* = 100 random iterations) were performed to determine whether the predictive accuracy of each epigenetic clock exceeded that expected by chance. For all models, observed predictive metrics significantly outperformed those obtained from permuted data (all *p* < 0.05). Predicted epigenetic age was calculated based on the combined methylation/entropy values of the selected adenines or cytosines, which were weighted by their respective coefficients.

### 4.5 Pharmacological Extension of Bee Lifespan

To test whether our epigenetic clocks track biological age, in addition to being highly predictive of chronological age, we sought to experimentally induce variations in biological age between individuals of the same chronological age. To this end, we used two well‐studied pharmacological agents that increase lifespan in a wide range of species—rapamycin and resveratrol—to increase adult male lifespan and assess how they influence epigenetic age. We proceeded in two consecutive steps.

First, we compared the lifespan and survival of rapamycin‐ or resveratrol‐treated versus control males to confirm whether these agents indeed prolong lifespan in *B. terrestris* males. Males were fed (i) sugar syrup solution containing DMSO (*n* = 49), which is the solvent used for rapamycin and resveratrol dilution, (ii) sugar syrup solution containing rapamycin (SelleckChem, S1039, final concentration = 100 μM, *n* = 32)—an inhibitor of mTOR, a protein complex that regulates growth, reproduction, and lifespan in diverse animal species (Saxton and Sabatini 2017), or (iii) resveratrol (MedChemExpress, HY‐16561, final concentration = 100 μM, *n* = 31)—an activator of the longevity‐promoting SIRT1 protein (Chen et al. 2020). Sugar syrup containing rapamycin or resveratrol was renewed three times per week to ensure optimal levels of drug activity. Treatment feeding started at 7 days old and continued until male death. The effects of rapamycin and resveratrol on survival curves were compared between treatment groups using a Cox proportional hazards model with mixed effects (*coxme* package in R (Therneau 2009)) in which experimental microcolony was computed as random factors.

Second, we tested if rapamycin and/or resveratrol affected the aging DNA methylome of *B. terrestris* males by sampling and sequencing 35‐day‐old males fed a control solution (sugar syrup), rapamycin or resveratrol (*n* = 3 per condition). DNA extraction, library preparation, ONT sequencing, differential methylation analysis, entropy quantification, and epigenetic age prediction were conducted as described for the initial dataset.

### 4.6 Statistical Analysis

All statistical tests were performed in the R programming environment (version 2023.06.0 + 421) (2023.06.0 + 421), except for differential methylation analyses which were conducted with Modkit in the Bash environment. Modkit is a ready‐to‐implement tool from ONT that uses a Bayesian framework to estimate differential base modification (e.g., methylated vs. unmethylated) between conditions. It uses a beta distribution to generate a posterior probability of modification at each site, then derives a Maximum A Posteriori (MAP)‐based *p*‐value which quantifies the confidence in observed differences. The MAP‐based *p*‐value considers effect size and coverage from Modkit pileup‐generated files to ensure statistical robustness of site‐resolution differential methylation detection (https://nanoporetech.github.io/modkit/dmr_scoring_details.html). False discovery rate (FDR) correction to control type I error was implemented using the Benjamini‐Hochberg method. Sites were considered as statistically differentially methylated (DMS) when FDR < 0.05.

Functional enrichment analyses were conducted using the R package topGO (Alexa and Rahnenfuhrer 2016) with the “weight01” algorithm, which considers term dependencies when performing statistical analyses, thus reducing redundancy and, consequently, minimizing false positives while retaining biologically relevant information.

Methylomic entropy was calculated by averaging mean per‐site methylation entropy to provide a single entropy value per sample (Hannum et al. 2013). Methylomic entropy was compared for 6 mA and 5 mC between the three age groups using one‐way ANOVA after confirming normality and homoscedasticity with Shapiro and Levene tests, respectively.

Rate of change (ROC) analyses were performed by fitting simple linear regression models to age‐related changes in 6 mA or 5 mC DMS. The slopes were derived to quantify the percentage methylation change per unit of time (i.e., weeks). Sample‐level mean ROC was calculated by averaging the slopes of statistically significant fitted models (*p* < 0.05 and *R* ^2^ > 0.65).

Epigenetic clocks were developed using penalized regression models. Briefly, samples were split into training (*n* = 10) and testing (*n* = 5) datasets with proportions roughly equal to 65% and 35% of the total dataset (*n* = 15). Given our limited sample size, we chose to remove sites missing methylation values, rather than applying imputation methods, to minimize the risk of overfitting resulting from introducing artificial artifacts. The glmnet R package (Friedman et al. 2010) was used to regress methylation matrices against known ages in the training set, then applying the model to the naïve (testing) dataset to assess model performances. Elastic net and ridge regressions were implemented by setting the alpha hyperparameter to 0.5 or 0, respectively. Mean absolute error (MAE), mean relative error (MRE) and Pearson's correlation (corr) were used as model performance estimation metrics.

## Author Contributions

T.R. designed and performed the experiments, collected and analyzed the data, wrote the paper. M.B. participated in data analyses. S.A. contributed to the conception and overall management of the project, acquired the funding, corrected the paper.

## Funding

This work was supported by funding from the Belgian Fund for Scientific Research (FRS‐FNRS), in the form of grants #C/RFE24/0071 (to TR), FC 57457 (to MB), and PDR T.0010.24, and by funding from the *Action de Recherches Concertées*—ULB in the form of grant #ARC 2025–2028 (to SA). The funders had no role in study design, data collection and analysis, decision to publish, or preparation of the manuscript.

## Conflicts of Interest

The authors declare no conflicts of interest.

## Supporting information


**Table S1:** 6 mA DMS between 7‐days‐old and 35‐days‐old males.
**Table S2:** 5 mC DMS between 7‐days‐old and 35‐days‐old males.
**Table S3:** 6 mA BP terms enriched between 7‐days‐old and 35‐days‐old males.
**Table S4:** 5 mC BP terms enriched between 7‐days‐old and 35‐days‐old males.
**Table S5:** 6 mA and 5 mC DMS between 35‐days‐old control males and 35‐days‐old males fed rapamycin.
**Table S6:** 6 mA and 5 mC DMS between 35‐days‐old control males and 35‐days‐old males fed resveratrol.
**Table S7:** Enriched BP terms between 35‐days‐old control males and 35‐days‐old males fed rapamycin.
**Table S8:** Enriched BP terms between 35‐days‐old control males and 35‐days‐old males fed resveratrol.

## Data Availability

All data generated or analyzed during this study are included in this published article and its electronic Supporting Information. ONT sequencing data are accessible in the NCBI SRA database.
